# Characteristics and Treatment Outcomes of Retreatment Tuberculosis Patients in Benin

**DOI:** 10.1155/2016/1468631

**Published:** 2016-03-24

**Authors:** Serge Ade, Omer Adjibodé, Prudence Wachinou, Narcisse Toundoh, Bérénice Awanou, Gildas Agodokpessi, Dissou Affolabi, Gabriel Adè, Anthony D. Harries, Séverin Anagonou

**Affiliations:** ^1^National Tuberculosis Programme, 01 BP 321 Cotonou, Benin; ^2^Faculté de Médecine, Université de Parakou, Parakou, Benin; ^3^International Union against Tuberculosis and Lung Disease, Paris, France; ^4^London School of Hygiene & Tropical Medicine, London, UK

## Abstract

*Objective*. To determine among retreatment tuberculosis patients in Benin baseline characteristics, culture, and drug sensitivity testing (DST) results and treatment outcomes.* Materials and Methods*. A retrospective national cohort study of all retreatment tuberculosis patients in Benin in 2013 using registers and treatment cards.* Results*. Of 3957 patients with tuberculosis, 241 (6%) were retreatment cases. Compared to new pulmonary bacteriologically confirmed tuberculosis (NPBCT) patients, there were significantly higher numbers of males (*P* = 0.04), patients from “Atlantique-Littoral” (*P* = 0.006), patients aged 45–64 years (*P* = 0.007), and HIV-positive patients (*P* = 0.04) among those retreated. Overall, 171 (71%) patients submitted sputum for DST, of whom (163) 95% were positive for* Mycobacterium tuberculosis* on Xpert MTB/RIF and/or culture and 17 (10%) were rifampicin resistant (9 with MDR-TB and 8 monoresistant to rifampicin). For those without MDR-TB (*n* = 224), treatment success was 93%. Worse outcomes occurred in those with unknown HIV status (RR: 0.27; 0.05–1.45; *P* < 0.01) while better outcomes occurred in those who relapsed (RR: 1.06, 95 CI: 1.02–1.10, *P* = 0.04).* Conclusion*. In 2013, a high proportion of retreatment patients received DST. Treatment success was good although more needs to be done to systematically increase the final follow-up smear examination. Reasons of high losses to follow-up from “Oueme-Plateau” should be investigated.

## 1. Introduction

Patients with retreatment tuberculosis (TB) represent those who have been treated previously for one month or more with anti-TB drugs and who have been diagnosed once again with the disease. These patients mainly include relapses, treatment after failure, or loss to follow-up on a first-line treatment regimen [1]. The number of these patients is not negligible. In 2014, of the 6.3 million TB cases that were notified by National TB Programmes (NTPs) to the World Health Organization (WHO), approximately 700,000 patients were already previously treated [2].

Interest in this category of TB comes from the fact that patients are known to have a higher risk of drug resistance compared with new cases. Therefore, in addition to recommendations that all TB patients be screened for Human Immunodeficiency Virus (HIV) coinfection, NTPs have been advised since 2010 by WHO to systematically test at diagnosis all retreatment TB patients for culture and drug susceptibility (DST with genotypic and/or phenotypical methods), in order to detect resistance to rifampicin which is usually synonymous with multidrug resistance (MDR, strictly defined as resistance of* Mycobacterium tuberculosis* to both rifampicin and isoniazid) [3, 4]. Unfortunately, there is concern about implementation of this recommendation as only 58% of all retreatment TB patients in the world were tested for drug resistance in 2014 [2]. Improving this proportion in the future requires an analysis of the situation at country levels.

In Benin, a sub-Saharan country that reports approximately 40 incident TB cases per 100,000 people per year, retreatment patients account for less than 10% of all notified TB cases. Unfortunately, there is dearth of information on the epidemiological characteristics and HIV status of these patients in annual reports in the country [5]. Two previous studies, which were only conducted in Cotonou, the economic capital, and which had included retreatment patients diagnosed in the periods of 1992–2001 and 2005–2009, respectively, reported a high loss to follow-up of 12%, with a treatment success of retreatment cases significantly lower than new cases. Moreover, the majority of these patients recorded as “loss to follow-up” during the retreatment initially had also defaulted from their first-line treatment [6, 7]. DST in retreatment cases was also only performed in early 2003 in those patients registered in Cotonou that also houses the “Laboratoire de Référence des Mycobactéries (LRM)”.

The current study was therefore undertaken in the whole country to determine the principal characteristics of retreatment patients, to assess how well DST was performed in this category of patients and whether there was any change in their treatment outcomes. Specific objectives were to determine among retreatment TB patients diagnosed and treated in 2013 in Benin: (i) the epidemiological, clinical and geographical characteristics and HIV status of these patients and compared to those registered with New Pulmonary Bacteriologically Confirmed TB (NPBCT); (ii) the proportion with DST results available along with the proportion showing resistance to rifampicin; (iii) treatment outcomes of these patients compared with NPBCT cases; and (iv) factors associated with a successful treatment outcome.

## 2. Materials and Methods

### 2.1. Study Design

This was a retrospective cohort study using routinely collected data.

### 2.2. General Setting and Study Sites

*Country*. Benin is a small low-income country in West Africa with a population of 10,315,244 inhabitants in 2015 [8]. The country shares borders with Burkina Faso and Niger in the north, Togo in the west, and Nigeria in the east. Benin is a low-income country with a gross national income per capita of US$1780 in 2013 and an under-five mortality rate of 85 per 1000 live births [9].

*National TB Programme & Management of Patients with a Previous History of TB*. The country follows the WHO DOTS strategy for diagnosis and treatment of TB patients [4]. All patients with a previous history of TB and who return to the health facility for presumptive symptoms of the disease are requested to provide two sputum samples on two consecutive days for acid-fast bacilli microscopy. Sputum samples are routinely examined using auramine-phenol staining and fluorescence microscopy. The diagnosis is confirmed by a positive result for acid-fast bacilli on sputum smears. Sputa of all confirmed retreatment TB cases in the 57 Basic Management Units (BMUs) are then sent by laboratory technicians using cetylpyridinium chloride solution to the LRM for both Xpert MTB/RIF and culture on Lowenstein-Jensen media. All retreatment TB patients, except those diagnosed in Cotonou, start on a standardised regimen of retreatment [3]. When the result of Xpert MTB/RIF is available (this usually takes some days or weeks), those who are found sensitive to rifampicin continue the standardised regimen. On the other hand, patients with resistance to rifampicin are switched to a MDR-TB treatment regimen.

Because of the closeness of the LRM, patients treated in Cotonou do not start TB treatment unless their Xpert MTB/RIF is available. This takes approximately 24 hours. The standardised retreatment regimen consists of an initial phase of rifampicin, isoniazid, ethambutol, and pyrazinamide for 3 months (with streptomycin added during the first 2 months) followed by a continuation phase using the first three anti-TB drugs for 5 months [3]. The treatment is strictly directly observed during the initial phase. Patients with resistance to rifampicin are treated with a shortened 9-month regimen recommended by the International Union against Tuberculosis and Lung Disease [10]. During retreatment of rifampicin sensitive patients, sputum samples are collected at 3 months, at 5 months, and at the end of the treatment for microscopy. Treatment is monitored through the use of treatment cards and registers. A quarterly supervision of all the 57 BMUs is systematically organized by the TB control programme coordination. All retreatment TB patients are systematically offered HIV testing at diagnosis. Those who are found coinfected receive co-trimoxazole, and Antiretroviral Therapy (ART) is provided within 2 weeks to 2 months after TB treatment initiation [11].

All molecular tests, culture, DST, TB treatment, co-trimoxazole, and ART are provided free of charge. Anti-TB and ARV drugs are not available in private pharmacies.

### 2.3. Study Population

All retreatment TB patients diagnosed and treated between January and December 2013 were included in the study.

### 2.4. Data Variables, Sources of Data, Data Collection Tools, and Definition of Variables

For each retreatment TB case, data were collected on epidemiological characteristics, region, HIV status, type of retreatment, ART status for HIV-positive patients, Xpert MTB/RIF results, culture and DST results, and treatment outcomes. Sources of data were TB registers and TB treatment cards. Xpert MTB/RIF, culture, and phenotypical DST results were extracted from a laboratory Excel file. For comparison, aggregate data of NPBCT patients were collected from the annual report. Data on patients were collected into a paper based questionnaire. Because of the retrospective nature of this study, data validation was not possible. The definitions of the NPBCT, the different types of previously treated patients, and the treatment and outcomes are explained in Table 1.

### 2.5. Analysis and Statistics

Data from the questionnaire were double entered into an electronic file using EpiData software and analysed using this software (EpiData version 3.1 for entry and version 2.2.2.182 for analysis, EpiData Association, Odense, Denmark). Data were analysed by using frequencies and percentages. Comparisons between categorical variables were done using the chi-square test and risk ratios as appropriate with 95% confidence intervals. Levels of significance were set at 5%.

## 3. Results

In 2013, 241 retreatment TB patients were diagnosed and treated in Benin on the basis of a positive result for acid-fast bacilli using sputum smear microscopy. They represented 6% of the 3957 total TB cases notified in the country in the same time-period. Demographic characteristics and HIV status of these patients were compared to those reported from NPBCT and are shown in Table 2. There were significantly more males (*P* = 0.04), patients from the “Atlantique-Littoral” (*P* = 0.006), patients aged between 45 and 64 years (*P* = 0.007), and HIV-positive patients (*P* = 0.04) among retreatment TB cases compared to NPBCT patients. On the other hand, patients younger than 24 years were significantly less common among previously treated patients (*P* < 0.001). Demographic characteristics and HIV status of the different types of retreatment TB cases are compared in Table 3. “Oueme-Plateau” reported significantly more “treatment after loss to follow-up cases” (*P* < 0.001) while “Atlantique-Littoral” reported more “relapse cases” (*P* = 0.04). In addition, significantly more males were found in “treatment after loss to follow-up” (*P* = 0.03). Otherwise, no significant differences were found.

The results of Xpert MTB/RIF and/or* Mycobacterium tuberculosis *culture among retreatment TB patients and rifampicin sensitivity are presented in Table 4. Overall, 71% of the 241 retreatment TB patients submitted their sputum for DST, and 95% of these patients showed a positive result on Xpert MTB/RIF and/or culture for* M. tuberculosis.* Of these patients, 17 (10%) were found to have resistance to rifampicin (9 with MDR-TB and 8 with monoresistance to rifampicin) and they were redirected to a MDR-TB treatment regimen.

Treatment outcomes of the remaining 224 retreatment TB patients were compared with those for NPCBT cases and the results are shown in Table 5. In 2013, the success rate of these patients was 93%. There were significantly more patients who completed their treatment without achieving the last follow-up examination, but otherwise there were no other differences between the groups. Factors associated with a successful outcome are presented in Table 6. Patients with relapse TB had a significantly higher chance of successful treatment outcome (RR: 1.06, 95 CI: 1.02–1.10, *P* = 0.04) while those with an unknown HIV status had a worse treatment outcome (RR: 0.27; 0.05–1.45; *P* < 0.01). In this cohort, in which all but one of the HIV-positive patients were on ART, no significant difference in treatment success was found between HIV-positive and HIV-negative patients.

## 4. Discussion

The main findings of this study were the predominance of males, adults aged between 45 and 64 years, and HIV-positive status among retreatment TB cases compared with NPBCT patients. Most of these previously treated TB patients were reported from “Atlantique-Littoral” where there were also predominately “relapse cases.” The large majority of patients who were reported from “Oueme-Plateau” were recorded as “lost to follow-up” prior to treatment, which is a department close to “Atlantique-Littoral” in the southern part of the country. Rifampicin sensitivity-status was known in nearly three-fourths of patients with one-tenth of those tested having resistance to rifampicin and therefore requiring a MDR-TB treatment regimen. Treatment outcomes of patients who were found without resistance to rifampicin were excellent, although some did not achieve the last follow-up sputum smear examination after treatment completion. Patients who relapsed had a greater chance of a successful treatment outcome, while those with unknown HIV status had worse treatment outcomes.

The strengths of this study were the inclusion of all retreatment TB patients diagnosed in the country, and therefore there was no need for any sampling framework. The study report also followed Strengthening the Reporting of Observational Studies in Epidemiology (STROBE) guidelines [12]. Data used for this study had been checked and validated during regular quarterly supervision of the 57 BMUs from the central unit. Limitations include the operational nature of the study and the use of routine data from registers and quarterly reports. Some information such as time to relapse after a previous treatment is often missed and therefore could not be included in the study.

This is the first assessment one year after the NTP coordination decided as a policy to perform DST at diagnosis for all retreatment cases countrywide. Benefits from the implementation of such a recommendation can be seen from this study. MDR-TB patients can promptly start appropriate treatment without having to start and fail a retreatment regimen, which has individual benefits as well as preventing further transmission of drug-resistant TB in the community. Studies from India have also shown that this approach improves treatment outcomes among previously treated patients [13]. According to our study findings, one-quarter of the patients did not achieve this goal of DST. The proportion of previously treated patients in whom DST has been performed largely varies according to the study settings, from less than 10% in the midwest Nepal to 91% in Sri Lanka [14, 15].

One of the major challenges in achieving DST for all patients that arose from discussions with health workers and that has also been reported elsewhere is related to transportation issues of samples from laboratories in BMUs to the LRM. This is a key step for successful implementation of culture and DST, particularly in resources-limited countries. In Benin, several options of sample transportation have been tried. In the first option, sputum specimens once collected were appropriately packaged in specific boxes in BMUs; and, during quarterly supervisions, national teams had the opportunity to bring them back to the LRM for analysis with results communicated later to the TB health workers in the BMUs. The consequences of this approach, however, were the high likelihood of having a negative culture due to lengthy times between sputum collection and the plating of specimens on Lowenstein-Jensen culture media and the subsequent delays in diagnosing and treating patients requiring MDR-TB treatment. In the second option, laboratory technicians in BMUs were requested to bring to the LRM specimens from patients and they were reimbursed for transportation. Unfortunately the high cost of this option did not allow it to continue. The third option and the current strategy is to send sputum specimens as soon as these are collected to the LRM through public transportation with transportation charges immediately refunded at receipt in the LRM. This strategy seems to be working well and contributes to an increase in the proportion of retreatment TB patients being tested. For example, an assessment of the 2014 programme activities of diagnosis and treatment of TB by the International Union against Tuberculosis and Lung Diseases estimated that 85% to 92% of retreatment TB patients in that year submitted sputum specimens to the LRM (Unpublished data, V. Schwoebel, Benin; TB control Programme, Annual report number 30; International Union against Tuberculosis and Lung Diseases). Finally, the forthcoming decentralization of Xpert MTB/RIF machines in regional laboratories will undoubtedly increase the proportion of patients having sputum specimens tested and this should accelerate the achievement of 100% DST.

There are a few other points to note. The proportion of patients requiring a MDR-TB treatment regimen in the country in 2013 was not higher in comparison with previous years [16, 17]. A predominance of males among retreatment TB cases was found in this study and this has also been reported elsewhere, although the reasons for this are not known [18]. “Atlantique-Littoral,” which houses Cotonou, the economic capital and the city with the highest density of population, always reports the highest number of TB cases in the country; and we are not surprised that this is also the case for retreatment TB cases. In terms of treatment outcomes and in comparison with previous reports, there have been improvements. We noticed that patients who were recorded as “lost to follow-up” are no longer at higher risk of defaulting during their retreatment [6, 7]. The treatment success is also higher than that observed elsewhere [13]. Furthermore, of the different types of retreatment TB patients, the highest success rate was reported in those who relapsed. Possible reasons for better outcomes include the following: (a) this is a new exogenous infection with drug-susceptible* Mycobacterium tuberculosis *rather than reactivation of a drug-resistant strain and (b) there is more awareness about TB symptoms and treatment management from the first experience with the disease resulting in earlier diagnosis. A worse treatment outcome was also reported in patients with unknown HIV status. In this group, it is possible that an HIV-positive status was known but the patient attempted to hide it with a consequent lack of appropriate HIV-care delivery.

The study has some implications for the programme. First, since the large majority of “loss to follow-up” patients retreated were from a specific region, there is a need to address the reasons why these patients stopped their first line treatment. Second, although the treatment success was good, we need to be stricter on ensuring that the last follow-up sputum examination is performed as these patients are being treated for the second time and are potentially at high risk of drug resistance. Third, although the proportion of TB patients with known HIV status is high (96%) in the country [5], it is important to try to screen all TB cases because of the poor treatment outcomes in those with unknown HIV status.

## 5. Conclusion

In 2013, the proportion of retreatment TB patients tested for culture and DST in Benin was 71%. Although the treatment success of these patients was encouraging, there is room for improvement. There is a need to increase the proportion of patients having the last follow-up sputum examination and to understand why one of the regions of the country has a sizeable number of patients recorded as “lost to follow-up.”

## Figures and Tables

**Table 1:** New category of patients and treatment outcomes recommended by the WHO [1].

	Definitions
*Category of TB patients*	
New pulmonary bacteriologically confirmed patients	Patients never treated for TB (or treated for less than 1 month) and whose sputum is positive by smear microscopy, culture, or WHO-approved Rapid Diagnostic (such as Xpert MTB/RIF)
Previously treated patients	Patients have received 1 month or more of anti-TB drugs in the past
Relapse patients	Patients have previously been treated for TB, were declared *cured *or *treatment completed *at the end of their most recent course of treatment, and are now diagnosed with a recurrent episode of TB
Treatment after failure patients	Patients have previously been treated for TB and *failed treatment *during or at the end of their most recent treatment course
Treatment after loss to follow-up patients	Patients have previously been treated for TB and were declared *lost to follow-up *during or at the end of their most recent course of treatment
Other previously treated patients	Patients have previously been treated for TB but their outcome after their most recent course of treatment is unknown or undocumented
Patients with unknown previous TB treatment history	Patients do not fit into any of the categories listed above

*Treatment outcomes*	
Cured	A pulmonary TB patient with bacteriologically confirmed TB at the beginning of treatment who was smear- or culture-negative in the last month of treatment and on at least one previous occasion
Treatment completed	A TB patient who completed treatment without evidence of failure, but with no record to show that sputum smear or culture results in the last month of treatment and on at least one previous occasion were negative, either because tests were not done or because results were unavailable
Treatment failed	A TB patient whose sputum smear or culture is positive at month 5 or later during treatment
Died	A TB patient who dies for any reason before starting or during the course of treatment
Loss to follow-up	A TB patient who did not start treatment or whose treatment was interrupted for 2 consecutive months or more
Not evaluated	A TB patient for whom no treatment outcome is assigned; this includes cases “transferred out” to another treatment unit as well as cases for whom the treatment outcome is unknown to the reporting unit
Treatment success	The sum of *cured *and *treatment completed *

**Table 2:** Demographic characteristics and HIV status of new pulmonary bacteriologically confirmed and retreatment tuberculosis patients, Benin, 2013.

	NPBCT^*∗*^	Retreatment TB	*P* value
Total	3129	241	
Sex			
Male	2063 (65.9)	175 (72)	0.04
Female	1066 (34.1)	67 (28)	0.04
Age (years)			
00–24	600 (19.2)	21 (8.7)	0.00005
25–44	1705 (54.5)	136 (56.6)	0.55
45–64	676 (21.6)	70 (28.9)	0.007
65 and above	148 (4.7)	14 (5.8)	0.43
Region			
Atacora-Donga	199 (6.4)	14 (5.8)	0.72
Borgou-Alibori	231 (7.4)	13 (5.4)	0.24
Zou-Collines	416 (13.3)	33 (13.7)	0.86
Mono-Couffo	486 (15.5)	29 (12)	0.14
Oueme-Plateau	708 (22.6)	47 (19.5)	0.26
Atlantique-Littoral	1089 (34.8)	105 (43.6)	0.006
HIV status			
HIV positive	396 (13.5^*∗*^)	43 (18.2^*∗∗*^)	0.04
HIV negative	—	193 (82)	—
Unknown	—	5 (2.4)	—

Note: NPBCT: new pulmonary bacteriologically confirmed tuberculosis. TB: tuberculosis.

^*∗*^Of the 2934 new pulmonary bacteriologically confirmed tuberculosis patients who underwent the HIV test, 396 were found positive.

^*∗∗*^Of the 236 retreatment tuberculosis patients who underwent the HIV test, 43 were found positive.

**Table 3:** Demographic characteristics and HIV status of the different types of retreatment tuberculosis patients, Benin, 2013.

	Relapse	Failure	Treatment after loss to follow-up	*P* value
Total	147	78	16	
Sex				
Male	102 (69.4)	56 (71.8)	16 (100)	0.03
Female	45 (30.6)	22 (28.2)	—	0.03
Age (years)				
00–24	10 (6.8)	10 (12.8)	1 (6.3)	0.29
25–44	86 (58.5)	42 (53.8)	8 (50)	0.69
45–64	42 (28.6)	22 (28.2)	6 (37.5)	0.74
65 and +	9 (6.1)	4 (5.1)	1 (6.3)	—
Regions				
Atacora-Donga	8 (5.4)	6 (7.7)	0	—
Borgou-Alibori	7 (4.8)	6 (7.7)	0	—
Zou-Collines	16 (10.9)	16 (20.5)	1 (6.3)	0.09
Mono-Couffo	16 (10.9)	12 (15.4)	1 (6.3)	0.46
Oueme-Plateau	27 (18.4)	10 (12.8)	10 (62.5)	0.00002
Atlantique-Littoral	73 (49.7)	28 (35.9)	4 (25)	0.04
HIV status				
HIV positive	27 (18.4)	14 (17.9)	2 (12.5)	0.84
HIV negative	116 (78.9)	63 (80.8)	14 (87.5)	0.70
Unknown	4 (2.7)	1 (1.3)	0	—

**Table 4:** Retreatment tuberculosis patients, drug susceptibility testing achieved, and bacilli sensitivity to rifampicin, Benin, 2013.

Total retreatment TB patients	241
Patients whose specimens were sent to the LRM for DST tests (*∗*)	171 (71)
MTB identification after culture and or Xpert MTB/RIF^§^	163 (95)
Xpert (+) Culture (+) (§)	86 (50)
Xpert (+) Culture (−) (§)	72 (42)
Xpert (−) Culture (+) (§)	5 (3)
Resistance to rifampicin (§)	17^¥^ (10)

Note: TB: tuberculosis; LRM: “Laboratoire de Références des Mycobactéries”; DST: drug susceptibility testing.

^*∗*^The percentage was derived from the total tuberculosis patients retreated.

^§^The percentage was derived from the total tuberculosis patients whose sputa were sent and analysed in LRM.

^*¥*^Of the 17 patients with resistance to rifampicin, there were 9 multidrug resistant and 8 monoresistant.

**Table 5:** Treatment outcomes of new pulmonary bacteriologically confirmed and retreatment tuberculosis patients, Benin, 2013.

	NPBCT^*∗*^	Retreatment TB	*P* value
Total	3124^§^	224^*£*^	
Successful outcome	2803 (89.7)	208 (92.9)	0.12
Cure	2587 (82.8)	184 (82.1)	0.79
Completion	216 (6.9)	24 (10.7)	0.03
Unsuccessful outcome	321 (10.3)	16 (7.1)	0.13
Failure	91 (2.9)	3 (1.3)	0.16
Death	170 (5.4)	9 (4)	0.37
Loss to follow-up	45 (1.4)	3 (1.3)	0.83
Not evaluated	15 (0.5)	1 (0.4)	0.72

Note: TB: tuberculosis.

^*∗*^NPBCT: new pulmonary bacteriologically confirmed tuberculosis.

^§^Of the 3129 new smear positive TB patients treated, treatment outcomes were assessed for 3124 patients in 2013.

^*£*^Of the 241 retreatment tuberculosis patients treated, 18 were found multidrug resistant and did not continue the retreatment regimen.

**Table 6:** Factors associated with a successful treatment among retreatment tuberculosis patients, 2013, Benin.

	Successful outcome	Risk ratio	[95% CI]	*P* value
Sex				
Female	60/62 (97.8)	1		
Male	148/162 (91.4)	0.94	0.88–1.01	0.24
Age group				
00–24	19/19 (100)	1		
25–44	117/124 (94.4)	0.94	0.90–0.99	0.59
45–64	60/67 (89.6)	0.90	0.83–0.97	0.34
65 and +	12/14 (85.7)	0.86	0.69–1.06	0.17
Type of TB				
NPBCT	2803/3124 (89.7)	1		
Relapse	133/140 (95)	1.06	1.02–1.10	0.04
Failure	63/69 (91.3)	1.02	0.95–1.10	0.67
Treatment after loss to follow-up	12/15 (80)	0.89	0.69–1.15	0.19
HIV status				
HIV negative	169/181 (93.4)	1		
HIV positive	38/39 (97)	1.04	0.98–1.11	0.47
HIV status unknown	1/4 (25)	0.27	0.05–1.46	0.001
Region				
Atacora-Donga	13/14 (92.9)	1.01	0.86–1.18	1
Borgou-Alibori	9/11 (81.8)	0.89	0.67–1.10	0.26
Zou-Collines	28/30 (93.3)	1.02	0.91–1.14	1
Mono-Couffo	27/28 (96.4)	1.05	0.96–1.15	0.68
Oueme-Plateau	40/42 (95.2)	1.04	0.95–1.13	0.72
Atlantique-Littoral	91/99 (91.9)	1		

Note: NPBCT: new pulmonary bacteriologically confirmed tuberculosis; TB: tuberculosis.
